# Screening for chronic kidney disease in rural Malawi: results from a diabetic clinic

**DOI:** 10.1186/s13104-019-4415-9

**Published:** 2019-07-01

**Authors:** Chiyembekezo Kachimanga, Richard Kamwezi, Emily B. Wroe, Lawrence Nazimera, Enoch Ndarama, Limbani Thengo, George C. Talama

**Affiliations:** 1 Partners In Health, Post Office Box 56, Neno, Malawi; 2 Ministry of Health, Neno, Malawi

**Keywords:** Renal impairment, Chronic kidney disease, Diabetes, Non-communicable disease, Malawi

## Abstract

**Objective:**

Patients with diabetes are at high risk of developing renal insufficiency and chronic kidney disease (CKD). As a result, screening for CKD is essential in diabetic patients as part of their care. This study investigated the prevalence of renal insufficiency, CKD, and correlates of CKD in diabetic patients attending Integrated Chronic Care Clinics in Neno District, Malawi.

**Results:**

Of 203 diabetic patients, 148 (73%) were screened for CKD by measurement of serum creatinine and urinary protein between April 2016 and January 2019. 39.2% (n = 58) of the patients had abnormal estimated glomerular filtration rate (eGFR), as estimated by CKD Epidemiology Collaboration formula and/or ≥ 2+ urine protein. 13.5% (95% CI 8.4–20.0%, 20/148) of the patients had renal insufficiency based on eGFR of less than 60 ml/min/1.73 m^2^. 8.8% (95% CI 4.8–14.6%, 13/148) had CKD based on eGFR of less than 60 ml/min/1.73 m^2^ measured twice at least 3 months apart. In bivariate analysis, CKD was associated with older age, high systolic blood pressure and lower fasting blood sugar. Despite the low sample size, the study showed a moderately high prevalence of renal insufficiency and CKD in a rural cohort of diabetic patients in Malawi.

## Introduction

The burden of chronic kidney disease (CKD) is increasing in Africa, in part due to the increased burden of non-communicable diseases (NCDs), in addition to the existing high burden of communicable diseases [1, 2]. Although the prevalence of CKD in the general population in Africa has recently been reported to be as high as 15%, the prevalence of CKD in diabetes mellitus patients is higher than in the general population; in a recent systematic review and meta-analysis, it is reported to be as high as 33% [3].

Due to differences in geographical areas, study population, definitions of CKD, and laboratory measurements, the prevalence and correlates of CKD in diabetic patients in sub-Saharan Africa varies widely between studies [2, 3]. For example, studies in Tanzania, Cameroon, Ethiopia, Ghana, and Botswana have reported CKD prevalence among patients with diabetes of 83.7%, 18.5%, 18%, 27% and 8.5% respectively [4–8].

In Malawi, the burden of NCDs continues to increase, with the prevalence of diabetes estimated to be as high as 6% in adults aged 15–64 years old [9, 10]. Despite evidence that diabetes is one of the main risk factors of CKD, there is paucity of data on CKD in diabetic patients in Malawi.

Routine screening for CKD in diabetic patients is a cost effective way of detecting CKD early [11], allowing for improved patient management, which may reduce the early onset of complications of CKD [12]. Additionally, screening prevents excessive costs when managing CKD complications [11]. Hence, screening for CKD was included in the package of interventions for NCD management in Malawi [13, 14]. However, most NCD clinics, especially in rural areas, do not provide CKD screening as they the lack necessary supplies and equipment [15, 16].

Due to differences in prevalence and correlates of CKD in other settings, it is important to investigate prevalence of CKD in Malawi, especially in rural Malawi where no data exists [17]. To fill this gap, this study reports on the prevalence of renal impairment, CKD, and correlates of CKD among patient with diabetes attending an NCD clinic in Neno District, Malawi between 2016 and 2019.

## Main text

### Setting

This study is a retrospective audit of diabetic patients enrolled in an Integrated Chronic Care clinic in Neno District, Malawi. Neno, located in the southwest zone of Malawi, is a rural and impoverished district, with a population of about 138,000 in 2018 [17]. Medical records of all diabetes mellitus patients aged 18 years and above who were screened for CKD at least once between April 2016 and January 2019 were retrospectively reviewed.

The Integrated Chronic Care clinic manages patients with HIV and/or a chronic NCD(s) (mainly: hypertension, diabetes mellitus, epilepsy and chronic respiratory disease), and is currently providing services in all 14 health facilities in Neno District. The clinics, managed by the Ministry of Health with support from a Non-Governmental Organisation called Partners In Health, are free-of-charge for all patients. Patients are followed every 1–3 months by a multidisciplinary team of midlevel providers, nurses and support staff [18]. By January 2019, the clinic had enrolled 8432 HIV and 3792 NCD patients, including 203 patients with diabetes (Partners In health internal data).

### Inclusion and exclusion criteria

All diabetes mellitus patients aged 18 years and above, enrolled in care for at least 3 months, with at least one visit in 2018, and at least one serum creatinine and/or urine protein result were included. Patients with a pre-existing diagnosis of CKD were excluded from the study.

### Data collection and laboratory measurements

Data was extracted from Neno District’s electronic medical record (EMR), which services all chronic conditions including HIV and NCDs. Clinic encounters in the Integrated Chronic Care Clinic are recorded on the Malawi Ministry of Health hypertension/diabetic master card, a standardised paper-based patient chart. At Neno the clinical information is replicated in the EMR.

All clinical and demographic measurements in the Integrated Chronic Care Clinic are protocol based, and these protocols are explained elsewhere [19]. For serum creatinine (in mg/dl), at-least 1 ml of venous blood was collected in heparinised bottles and analysis was done immediately with I-STAT point of care chemistry machine (Abbott, USA) using chem8+ cartridges. Where I-STAT machine were not available, the samples were immediately transported to Neno District Hospital laboratory and analysed using Mindray BS 120 machine. For urine dipstick, Test-10 It (Life assay diagnostics limited) urine test strips were used on freshly collected urine and the results read within 1 min. Urine protein results were coded as negative, trace, +1, +2 and +3.

### Measurements and outcomes

CKD was defined using Kidney Disease: Improving Global Outcomes (KDIGO) as an estimated glomerular filtration rate (eGFR) of less than 60 ml/min/1.73 m^2^ and/or urinary protein 2+ or more, measured on two occasions at least 3 months apart. Renal insufficiency was defined as eGFR less than 60 ml/min/1.73 m^2^ measured on one occasion only. The eGFR was estimated based on serum creatinine using Chronic Kidney Disease Epidemiology Collaboration (CKD-EPI) formula without using the factor of race. This formula was chosen as it has demonstrated better prediction of eGFR in Malawian adults in comparison to CKD-EPI with factor of race, Cockcroft-Gault, and Modification of Diet in Renal Disease 4 formulas [20]. EGFR was classified as normal (eGFR > 90 and/or urinary protein normal or less than 2+) or abnormal (stage 0, 1, 2, 3a, 4a, and 5) based on KDIGO guidelines [21].

Several other variables included in our analysis were extracted from routine patient encounters. These variable included:

- *Demographic characteristics* Age (years) and gender (male and female).
- *History and clinical encounter measurements* Type of diabetes (type 1 or type 2), number of years with diabetes and duration in clinic (< 1 year, 1–2 years, > 2 years), most recent fasting blood glucose (FBG) (in mg/dl), diabetes mellitus patients with good FBG (strict control: < 126 mg/dl, reasonable control: 126–180 mg/dl and poor control: over 180 mg/dl), most recent body mass index (BMI) (kg/m^2^), most recent diastolic blood pressure (DBP) and systolic blood pressures (SBP) (mmHg).
- *Diabetes mellitus co-morbidities* Current treatment for hypertension.

Some variables in our intended analysis had a high degree of missing data and variables that were missing in more than 90% of the participants were not included in the analysis; and these variables included history of smoking and alcohol intake and rare complications like stroke and cardiovascular diseases.

### Statistical analysis

All data were extracted to Microsoft Excel 2013 and exported to Stata version 14 for data cleaning and analysis. Missing data were re-collected using both electronic and physical patient charts.

Descriptive statistics were used to describe all the variables. Bivariate analysis was used to show differences between patients with and without CKD. For categorical variables, Chi2 test or Fishers exact test were used as appropriate. For continuous variables, t-test were used for normally distributed data and the Mann–Whitney test for non-normally distributed variables. p < 0.05 showed statistical significance.

### Results

A total of 148 diabetic patients, 16.9% type 1 (n = 25) and 83.1% type 2 (n = 123), were screened for CKD. This represented 73% of the 203 diabetic patients enrolled in care by January 2019 (Table 1). The mean age was 54 years, and about two-thirds of the patients (n = 77, 66.4%) were female. The majority of the patients had lived with diabetes and had attended the clinic for over 2 years (about 60.8% and 43.7% respectively).

**Table 1:** Characteristics of patients with diabetes

	All diabetic patients (N, %)	CKD (N/%)	No CKD (N/%)
Total patients screened	148	13 (8.8)	135 (91.2)
Demographic characteristics			
Age (mean, sd)^a^-years	53.8 (13.7)	63.9 (9.7)	52.8 (13.6)
Gender			
Female	98	8 (8.2)	90 (91.8)
Diabetes type			
Type 1 diabetes	25	2 (8.0)	23 (92.0)
Type 2 diabetes	123	11 (8.9)	112 (91.1)
Years since diagnosis of diabetes			
Less than 1 year	29	1 (3.4)	28 (96.6)
1–2 years	20	3 (15.0)	17 (85.0)
Over 2 years	76	7 (9.2)	69 (90.8)
Duration in clinic			
Less than 1 year	44	3 (6.8)	41 (93.2)
1–2 years	36	6 (16.7)	30 (83.3)
Over 2 years	62	4 (6.4)	58 (93.6)
Most recent fasting blood glucose (median, IQR)^a^-mg/dl	156.6 (115.2–234)	130.6 (99.9–151.1)	160.2 (121.0–249.0)
Patients with good glucose control (< 126 mg/dl)	39	5 (12.8)	34 (87.2)
Patients with reasonable glucose control (128 to < 180 mg/dl)	42	6 (14.3)	36 (85.7)
Initial creatinine (median, IQR)-mg/dl	0.7 (0.6–0.9)	1.4 (1.2–2)	0.7 (0.6–0.9)
Risk factors			
BMI (median, IQR)-kg/m^2^	26.5 (22.5–30)	26.5 (25.6–29.2)	26.5 (22.3–30.1)
BMI over 25 (median, IQR)-kg/m^2^	29.1 (27.3–32.9)	27.8 (26.0–31.6)	29.3 (27.3–33.2)
Most recent systolic blood pressure (median, IQR)^a^-mmHg	130 (117–145)	135 (132–154)	129 (116–142)
Most recent diastolic blood pressure (median, IQR)-mmHg	83 (77–89)	91 (81–97)	83 (77–88)
Diabetic co-morbidities and complications			
Hypertension	103	12 (11.7)	91 (88.3)

*CKD* chronic kidney disease, *%* percentage, *Sd* standard deviation, *IQR* interquartile range, *BMI* body mass index

^a^Significant difference between CKD and no CKD group

The majority of the patients were overweight (median BMI 26.5 kg/m^2^), had a mean most recent FBG of 156.6 mg/dl, and median most recent SBP and DBP of 130 mmHg and 83 mmHg respectively. 32.1% (n = 42) and 29.8% (n = 39) had reasonable and strict blood glucose control respectively. Hypertension was the main co-morbidity (n = 103, 69.6%).

The initial median creatinine and eGFR were 0.7 mg/dl (IQR 0.6–0.9) and 96.9 (IQR 77.1–110.5) respectively. 60.8% of patients (n = 90) had normal eGFR, 1.4% (n = 2) had stage G1, 25.7% (n = 38) had stage G2, and 12.3% (n = 18) had eGFR stage G3a to stage G5 (Table 2). 13.5% (95% CI 8.4–20.0%, n = 20) of the patients had renal impairment. After a minimum of 3 months, creatinine was repeated in 14 of the 20 patients with renal impairment. Only one patient had eGFR that had normalized, and 13 patients had eGFR less than 60 ml/min/1.73 m^2^. The remaining six patients could not be located. The prevalence of CKD in this cohort was 8.8% (CI 4.8–14.6%).

**Table 2:** Estimated GFR (eGFR) stages in all diabetic patients

Stages of eGFR	eGFR (ml/min/1.73 m^2^)	N (%)
Normal EGFR^a^	≥ 90	90 (60.8)
G1 (normal and urine protein 2+ or more)	≥ 90	2 (1.4)
G2 (mildly decreased)	60–89	38 (25.7)
G3a (mildly to moderately decreased)	45–59	9 (6.1)
G3b (moderately to severely decreased)	30–44	6 (4.1)
G4 (severely decreased)	15–29	2 (1.4)
G5 (kidney failure)	< 15	1 (0.7)
Total		148 (100)

^a^Patients in this category had eGFR over 90 and protein less than 2+

There were significant differences in age, most recent SBP, and most recent FBG between diabetic patients with CKD and without CKD. Diabetic patients with CKD were older (63.9 years versus 52.8 years, p = 0.005), and had higher most recent SBP (135 versus 129 mmHg, p = 0.004). However, patients with CKD had a lower most recent FBG than patients without CKD (130.6 vs. 160.2 mg/dl, p = 0.04). Although not statistically significant, most recent DBP and hypertension diagnosis were higher in the CKD group in comparison to those without CKD. No differences in the other risk factors were observed between the groups.

### Discussion

This is the first study to investigate renal insufficiency and CKD in a cohort of diabetic patients attending NCD clinics in rural Malawi. Almost two-thirds of the patients were females and this pattern of higher health service utilization by women is common in Malawi [22]. In Malawi, most of the studies evaluating renal impairment and CKD were done in the context of HIV, patients acutely admitted in hospitals, and in urban centers [23–28]. In this study, about 40% of the diabetic patients had abnormal eGFR, demonstrating the importance of CKD screening. Identifying these patients can potentially improve management by promoting strategies that will ensure protection of the kidneys, avoiding drugs that may cause further kidney damage and reduce progression of CKD and ultimately end stage renal disease. More importantly, screening allowed health care providers to target patients who needed repeat testing to monitor their kidney function and diagnose CKD.

The prevalence of CKD in diabetic patients was about 9%, lower than some recent estimates in Africa. Two recent systematic reviews and meta-analysis report the prevalence of CKD in diabetic patients at 22% and 25% in Africa [2, 3]. However, a study in Botswana found a similarly lower prevalence of CKD in diabetic patients (8.4%) [29]. The lower prevalence may be due to the use of KDIGO guidelines, which require two measurements at least 3 months apart.

In this study, increased age and higher SBP were associated with CKD. Other studies have found similar associations with CKD [8, 29, 30]. However, lower FBG was associated with CKD. Although this needs further investigation and was in a small sample size, we speculate that this may be the case as FBG varies on a day to day basis in comparison to glycosylated hemoglobin. Glycosylated hemoglobin may have been a good measure of control in this study.

The study has research and clinical implications. Neno District plans to continue screening for CKD and patients with severe CKD are now enrolled in an advanced NCD clinic for care. Based on findings of this study, diabetes clinics should invest in screening for CKD. Finally, we propose a repeat of the study in rural and urban sites with larger patient cohort to further inform clinical practice and policy.

### Conclusion

Although the sample size was small, we found moderately high renal insufficiency and CKD, 13.5% and a minimum of 8.9% respectively, in a diabetic cohort attending Integrated Chronic Care clinics in rural Malawi. In bivariate analysis, CKD was associated with older age, high SBP and lower FBG.

## Limitations

As a facility-based clinical audit that used routinely collected data;

- The study excluded all diabetic patients younger than 18 years, patients that were lost to follow up, and patients that have diabetes and are not getting care in Neno, Malawi.
- The data quality depended on how accurately the data was recorded in the chart.
- The study may not be representative of communities in Neno District and other districts of Malawi.
- The Correlates of CKD chosen for this study depended on the available data and may have excluded other risk factors.
- The study does not establish the temporal sequence between CKD and diabetes or any of the risk factors.

## Data Availability

All data used in this study belonged to Malawi Ministry of Health and hence cannot be made public. However, data can be provided by the corresponding author if its requested.

## Abbreviations

CKD: chronic kidney disease
NCD: non communicable diseases
KDIGO: Kidney Disease: Improving Global Outcomes
EGFR: estimated glomerular filtration rate
CKD EPI: chronic kidney disease epidemiology collaboration
BMI: body mass index
CI: confidence interval
EMR: electronic medical records
FBG: fasting blood glucose
SBP: systolic blood pressure
DBP: diastolic blood pressure
